# Supplementary material for: Heavy quasiparticles and cascades without symmetry breaking in twisted bilayer graphene
Source: Nat Commun. 2023 Aug 18;14:5036. doi: 10.1038/s41467-023-40754-4 (PMC10439139; doi:10.1038/s41467-023-40754-4)
Supplement: Supplementary file 1 — Supplementary Information [file 41467_2023_40754_MOESM1_ESM.pdf]

# SUPPLEMENTARY INFORMATION

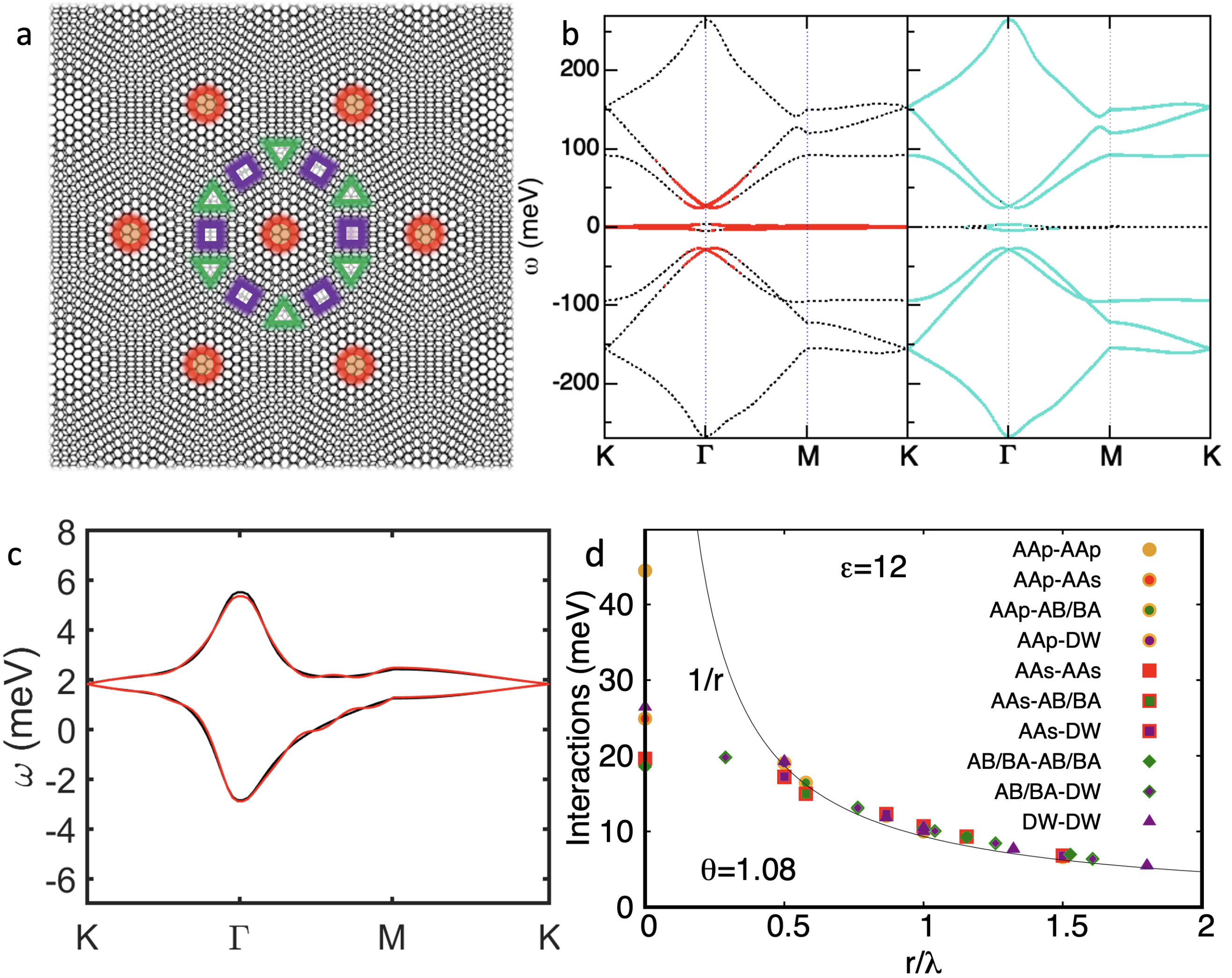


**Supplementary Figure** 1. **Model details** (a) Schematic plot of the triangular AA, honeycomb AB/BA, and kagome DW lattices where the eight orbitals per valley and spin are centered. Besides the two correlated $AA_p$ orbitals (orange), at the AA regions of the moiré unit cell another orbital with $s$ character $AA_s$ is centered (red). The model also includes two $p_z$ orbitals centered at the honeycomb lattice formed by the AB/BA regions (green) and three $s$ orbitals at the kagome lattice (purple) formed by domain wall DW regions separating two AB/BA points. (b) Orbital weight of the two correlated $AA_p$ (left) and the six less correlated orbitals (right) to the band structure of the eight orbital model used in the calculation. (c) Zoom of Fig.1a corresponding to the non-interacting flat band obtained from the continuum model (black) for an angle $\theta = 1.08°$ and its fitting with the eight orbital model. (d) Intra and inter-orbital density-density interactions as a function of the distance between the orbitals. Here $\lambda$ is the moiré unit cell.

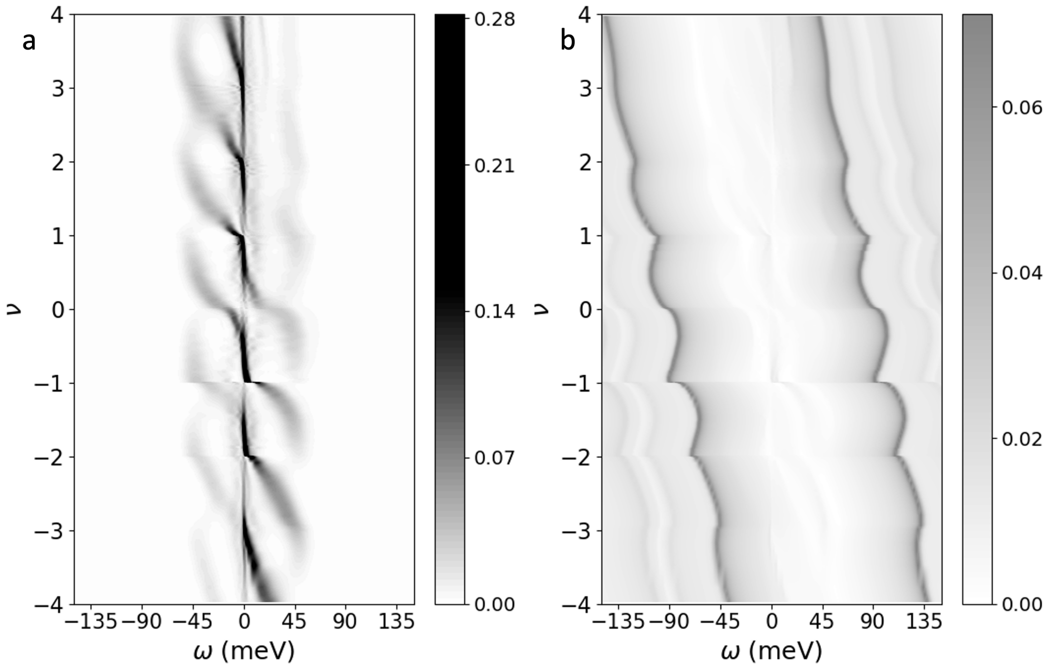


**Supplementary Figure** 2. **Contribution to the spectral weight of the two types of orbital.** Contribution to the density of states in Fig. 1b of the (a) two strongly correlated $AA_p$ orbitals. (b) Same as (a) but for the six less correlated $\mathrm{AA}_s$, AB / $\mathrm{BA}p_z$ and DW$s$ orbitals. The $lc$ contribution is reduced at small energies. Nevertheless some effects of the resets can be also appreciated in their spectral weight at low energies. The contribution of the $AA_p$ orbitals will be more visible in STM measurements at the AA site, while the spectral weight of the less correlated orbitals will have larger weight at the AB position (the $\mathrm{AA}_s$ has an annular shape). Due to the finite extension of the orbitals, this separation of the orbitals in spatial regions should not be taken too literal.

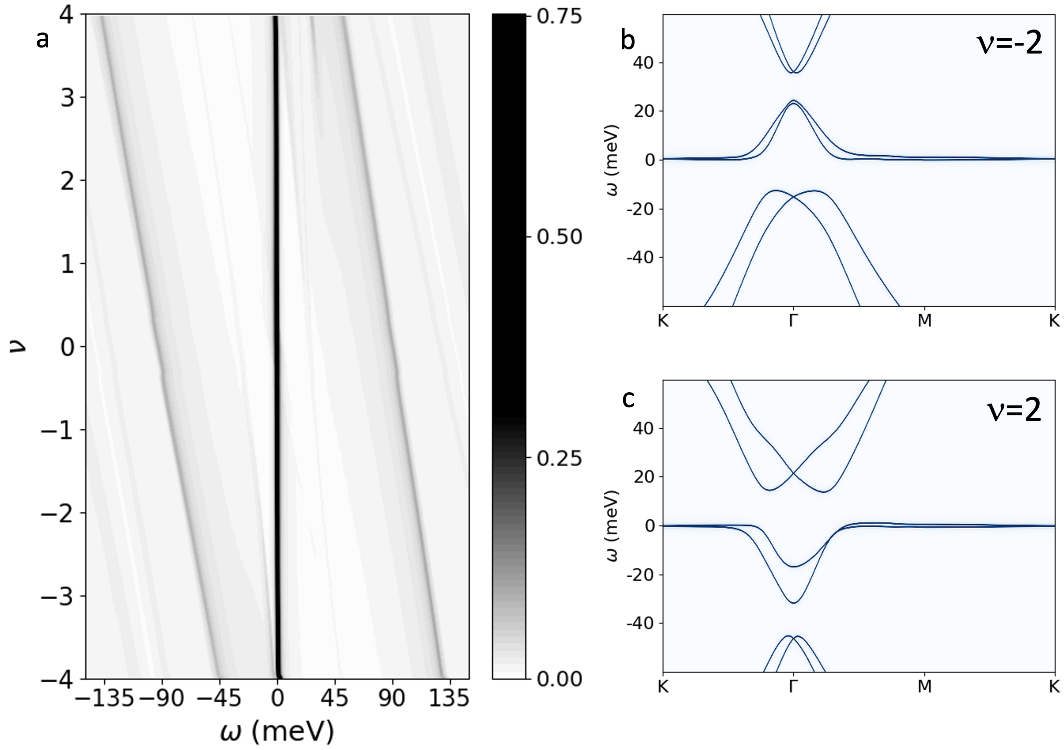


**Supplementary Figure** 3. **Density of states and band deformation in the Hartree approximation**. (a) Same as Fig. 1b but treating all the interactions in the Hartree approximation. The cascades of spectral weight and the oscillations in the remote band peaks have disappeared. These peaks shift approximately linearly with doping with a slope 11 meV per electron doped. (b) and (c) Low energy bands calculated for $\nu = -2$ and $\nu = 2$ in the Hartree approximation. The band shape changes[1–3] with respect to the non-interacting one in (d). Nevertheless, the effect of such changes on the spectral weight reorganization in (a) is small.

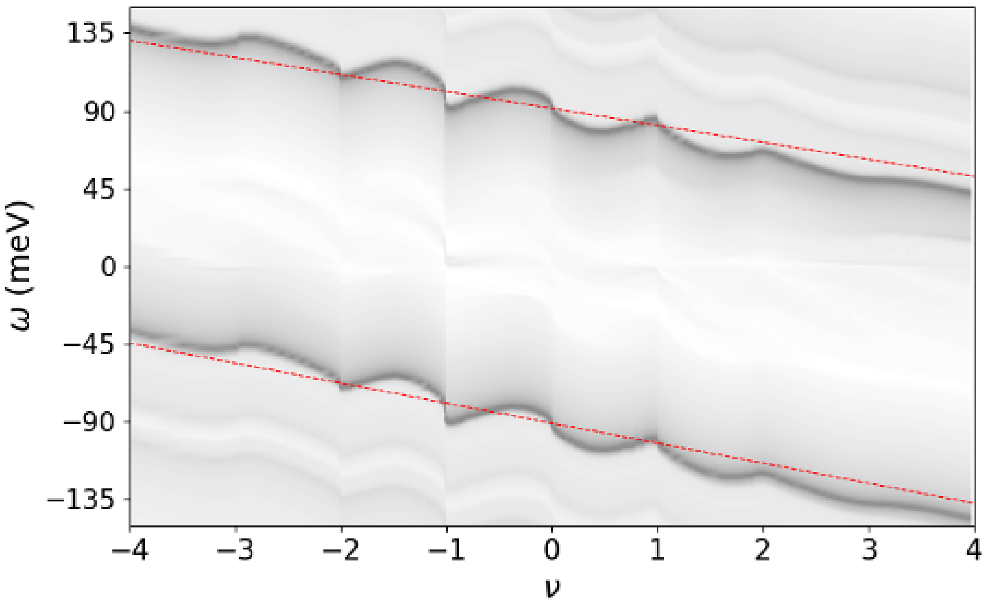


**Supplementary Figure** 4. **Oscillations of the remote band energies**. Dependence of the remote band peak energies as a function of doping. The red lines are an approximation to the linear shift of the peak positions in the Hartree approximation in Fig.3.

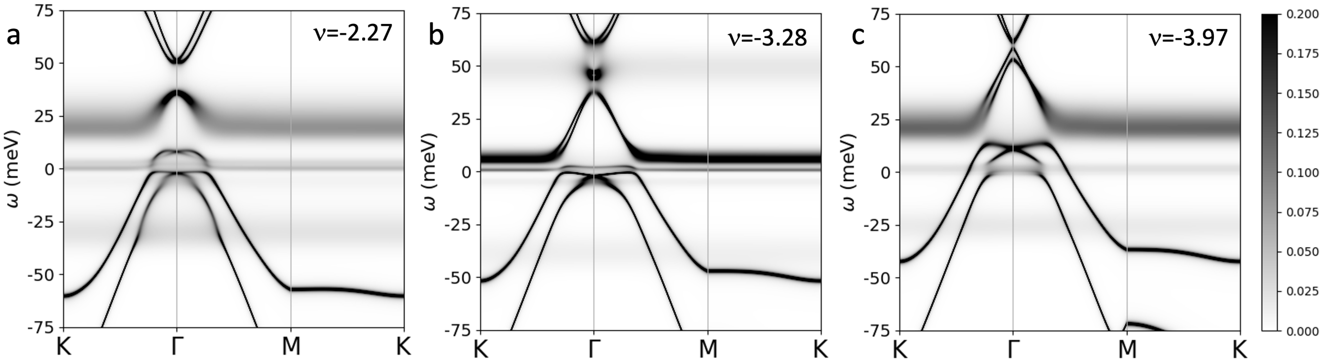


**Supplementary Figure** 5. **Momentum resolved spectral weight for** $|\nu| > 2$**.** Interacting bandstructure obtained from the DMFT+Hartree calculations for dopings $\nu < -2$. (a) $\nu = -2.27$, (b) $\nu = -3.28$ and (c) $\nu = -3.97$. It can be observed how the remote bands cross the chemical potential contributing significantly to the DOS at the Fermi level and to the doping dependence of the spectral weight. The contribution of the remote bands here resembles the one in charge transfer insulators. Due to this crossing at large dopings the filling of the $lc$ orbitals is less sensitive to the integer dopings in Fig. 3 and the oscillations of the remote band peak energies in Fig. 1 are not so well defined.

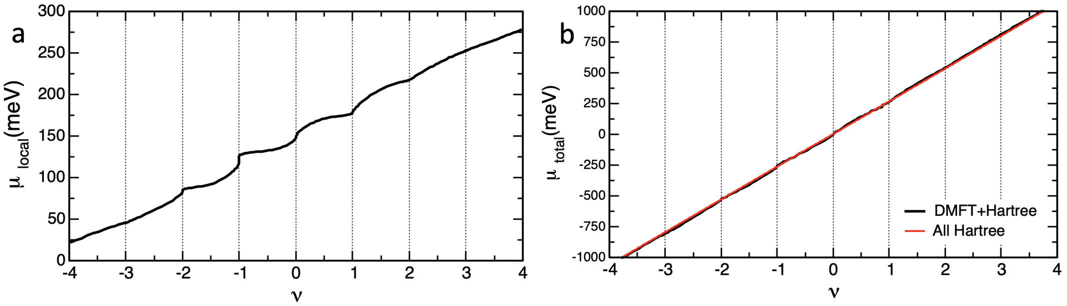


**Supplementary Figure** 6. **Chemical potential as a function of doping.** In our calculations we define two different chemical potentials with a one to one correspondence between both quantities. The local chemical potential $\mu_{local}$ in (a) enters into the DMFT calculation. Its value is primarily controlled by the interaction U and the filling of the $AA_p$ orbitals. In (b) The global chemical potential $\mu_{total}$ enters in the Hartree part of the DMFT+Hartree calculation and serve to determine the onsite shifts of all the orbitals. It strongly depends on the long range interactions and account primarily for a global shift of all the bands. $\mu_{total}$ has a very large magnitude and an almost linear dependence on the density which closely follows the value obtained when all the interactions, including U, are treated at the Hartree level. In the DMFT+Hartree calculation $\mu_{total}$ has small steps at the integer dopings. The derivative of $\mu_{total}$ (not shown) is dominated by the linear dependence and the features around the integers are quite noisy.

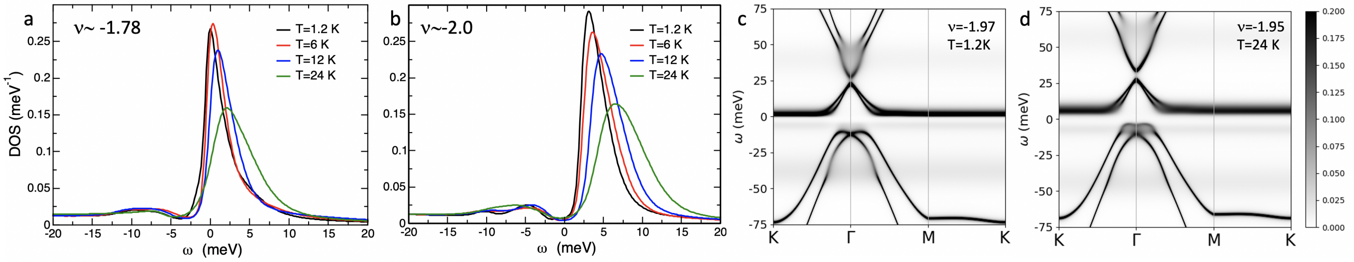


**Supplementary Figure** 7. **Temperature dependence of the spectral weight.** Dependence of the density of states on temperatures for dopings close to $\nu = -2$. (a) $\nu$=2 and (b) $\nu$ ≈1.77-1.78 showing a similar behavior as discussed in Fig. 3 for $\nu$ $-1.96$. With increasing temperature the peak shifts towards positive energies, its height decreases and the shape is slightly changed. The pseudogap around zero or small energies slightly decreases with decreasing temperature. (c) and (d) Band structure corresponding to the DOS in at T=1.2K and T=24K discussed in Fig. 3. With increasing temperature, the bands become more incoherent and small band shifts are observed.

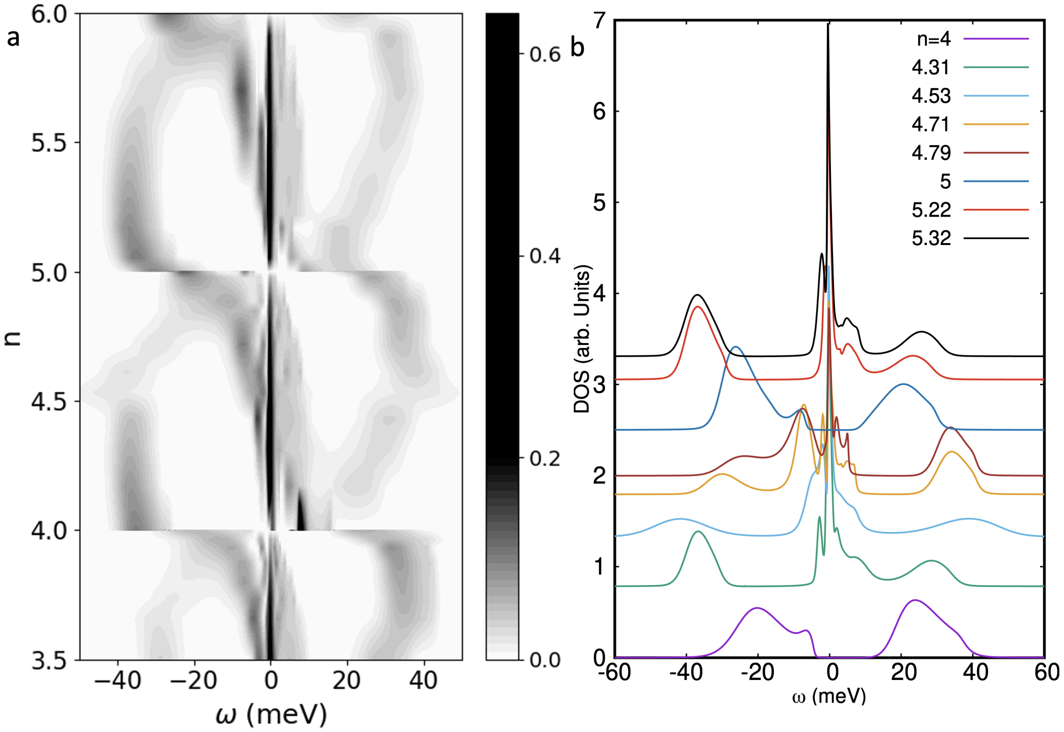


**Supplementary Figure** 8. **Density of states of an only AAp model.** (a) Colour plot corresponding to the density of states calculated with DMFT of a Hubbard model containing only the $AA_p$ orbitals (four orbital model degenerate in spin) interacting with both intra and inter-orbital interaction U=44.5 meV, as in the eight orbital model, for fillings $n$ within the range $-3.5 < n < 6$. Here half-filling (the equivalent for this model to the CNP) is 4. A clear reorganization of the spectral weight with doping extending up to energies of order U is observed. At each integer the lower (upper) Hubbard band approaches the chemical potential at integer fillings if the system is doped with holes (electrons). Strong resets in the spectral weight are found associated to the gaps at integer fillings. At non-integer fillings a large density of states associated to the formation of a heavy quasiparticle, typical of doped Mott insulators, is found. (b) Line cuts of the density of states for selected dopings. Curves are shifted for clarity. The typical three peak structure is observed at partial non- integer fillings, being the thin peak at zero energy the quasiparticle peak and the two broad peaks at a few tens meV the Hubbard bands. The Hubbard bands shift with doping. At integer values there are large Mott gaps and resets in the Hubbard bands.

## SUPPLEMENTARY REFERENCES


[1] L. Rademaker and P. Mellado, Charge-transfer insulation in twisted bilayer graphene, Phys. Rev. B **98**, 235158 (2018).

[2] F. Guinea and N. R. Walet, Electrostatic effects, band distortions, and superconductivity in twisted graphene bilayers, Proceedings of the National Academy of Sciences **115**, 13174 (2018).

[3] M. J. Calderón and E. Bascones, Interactions in the 8-orbital model for twisted bilayer graphene, Phys. Rev. B **102**, 155149 (2020).
